# Effects of antimony on redox activities and antioxidant defence systems in sunflower (*Helianthus annuus* L.) plants

**DOI:** 10.1371/journal.pone.0183991

**Published:** 2017-09-05

**Authors:** Alfonso Ortega, Inmaculada Garrido, Ilda Casimiro, Francisco Espinosa

**Affiliations:** FBCMP Research Group, University of Extremadura, Campus Avenida Elvas, Badajoz, Spain; National Botanical Research Institute CSIR, INDIA

## Abstract

The alterations induced by the toxicity of antimony (Sb) in the roots and leaves of sunflower plants were determined. The plants were grown hydroponically with different concentrations of Sb, a heavy metal which reduces biomass production and growth. There was preferential accumulation of Sb in the tissues of the roots, with the concentrations in the leaves being much lower. The accumulation of other mineral elements was also altered, especially that of Fe and Zn. Chlorophyll content declined, as also did the photosynthetic efficiency, but the carotenoid content remained unaltered. The total content of phenolics, flavonoids, and phenylpropanoid glycosides rose, evidence of their participation in the defence response. Increases were observed in the amount of superoxide anion in both roots and leaves, and in lipid peroxidation levels, especially with the highest Sb concentration of 1.0 mM. The induced oxidative stress leads to a strong increase in the SOD, POX and APX antioxidant activities, while the GR activity was only increased in the leaves and at the 1.0 mM Sb concentration. In contrast, the DHAR activity increased considerably in both organs. The GSNOR activity increased only in roots, and the total RSNOs increased. The total amount of AsA + DHA increased in roots and remained unaltered in leaves, whereas that of GSH + GSSG decreased considerably in all cases. As a whole, these results are evidence for the development of a strong oxidative stress induced by Sb, with there being a clear imbalance in the content of the compounds that constitute the AsA/GSH cycle. 0.5 mM Sb enhances GST expression, especially in leaves. This, together with the increase that was observed in the amount of GSH, may play an important part in detoxification. This oxidative stress affects both the phenolic and the ROS/RNS metabolic processes, which seems to implicate their involvement in the plant's defence and response to the stress.

## Introduction

Heavy metal and metalloid contamination represents a serious problem for the environment and agriculture as well as for human health. One of the most toxic elements is Antimony (Sb). The amount of this element found in the Earth's crust is low, between 0.2 and 0.3 mg kg^-1^ [1]. In soils, its concentration ranges from 0.3 to 8.6 mg kg^-1^ [2]. However, the growth in human activities such as mining, smelting, traffic, and industry has led to a considerable increase in its accumulation in soils [3,4]. Soil Sb levels greater than 1800 mg kg^-1^ have been measured around mines [5–7]. In this sense, Sb contamination is a serious problem today, with this element being one of the main contaminants [4]. The European Union and the World Health Organization consider antimony, together with arsenic, as contaminants that call for greater attention because of their dangerousness as agents that induce cancers and cardiovascular, liver, and respiratory diseases [8,9]. Antimony has an atomic weight of 121.76, and is a trace element that is not essential for plants, but which can be absorbed by them. In mining and industrial areas, a strong presence of Sb has been observed in the soil, with large accumulations in plants [5,6,10–15]. Sb is extensively accumulated in root tissues [16,17], although other studies describe a greater accumulation in leaves in different species [6,18,19]. Some plants have been described as hyperaccumulators of Sb [6,13,20,21].

Plants subjected to heavy metal stress show a primary response consisting of the rapid production of reactive oxygen species (ROS) and reactive nitrogen species (RNS) [22]. The ROS include superoxide anion (O_2_·^-^), hydrogen peroxide (H_2_O_2_), hydroxyl anion (OH^-^), and singlet oxygen (^1^O_2_) [23,24]. The RNS include nitric oxide (NO), peroxynitrite (ONOO^-^), S-nitrosothiols (RSNOs), and S-nitrosoglutathione (GSNO) [25]. The RBOH-NOX and peroxidases bound to the cell wall are the O_2_·^-^ and H_2_O_2_ generating systems in the plasmalemma and apoplast. They begin the oxidative shock, which also occurs in peroxisomes, mitochondria, and chloroplasts [22,23]. The rapid and strong production of ROS and RNS constitutes a nitrosative shock to which the plant responds with antioxidant systems, both enzymatic and non-enzymatic, responsible for controlling the levels of the reactive species, and therefore for the redox homeostasis which is crucial for maintaining the functionality of biological systems [26–28]. Enzymes such as SOD, POX, glutathione reductase, and monodehydroascorbate reductase are involved in the enzymatic antioxidant systems. The non-enzymatic antioxidant systems include ascorbate and glutathione, as well as phenolic-type compounds such as flavonoids, phenylpropanoids, glycosides, and carotenoids [29]. Ascorbate is considered a strong antioxidant because of its ability to intervene in redox reactions, and it plays a crucial part in the system for the removal of H_2_O_2_ via the AsA-GSH cycle [27,30,31]. In this cycle, APX uses two molecules of AsA to reduce H_2_O_2_, generating water and MDA. The MDA dismutates to DHA, and subsequently regenerates AsA through MDHAR. Glutathione plays an important role in the protection against oxidative damage induced by ROS at the cellular level, and the GSH/GSSG balance is a key indicator of the cellular redox state [32]. It also intervenes in the AsA regeneration process, forming part of the AsA-GSH cycle [31]. By means of GSNOR, it can also react with NO forming S-nitrosoglutathione, and possibly the GSH/GSNO ratio is another key element in the control of the cell's redox state, connecting ROS and RNS [33]. Phenolic compounds also have strong antioxidant activities [34]. Thus, polyphenols can directly eliminate ROS, and can inhibit lipid peroxidation of membranes by scavenging lipid alkoxyl radicals [29]. Phenolic compounds, especially flavonoids, have a great capacity to modify membrane packing and fluidity [35]. These changes affect the ability of ROS to diffuse through the membranes, and in this way decrease the peroxidation reactions [36]. It has also been shown that flavonoids can directly eliminate ROS because of their ability to donate electrons or hydrogen atoms [35]. Flavonoids and phenylpropanoids are both oxidized by peroxidases (APX and POX), and thus act to eliminate H_2_O_2_ by means of a phenolic/ascorbate/peroxidase system [37]. Carotenoids are compounds capable of detoxifying various forms of ROS [38]. They contain a chain of isoprene residues with numerous conjugated double bonds which allow the capture of excited molecules such as ROS [23]. In addition to these ROS production and elimination systems, RNS are also involved in these processes, and they have been shown to participate in the response to heavy metal toxicity [39]. Overproduction of ROS and RNS can trigger what is called nitro-oxidative stress [33]. The reaction of O_2_·^-^ and NO forms peroxynitrite, which produces protein nitration and nitrosylation. Reduced glutathione and NO produce S-nitrosoglutathione, the form in which NO is accumulated in the cells. This GSNO can also produce protein nitration and nitrosylation, together with lipid nitration. The production of S-nitrosoglutathione (GSNO) and the activity of GSNOR appear to be key systems in the interrelationship of ROS and RNS and in the effects of NO on cells [33]. In sum, the production of ROS and their interactions with RNS seem to play a key role in triggering and controlling plants' defence response [40].

The stress induced in plants by high concentrations of Sb leads to different responses depending on the species studied. Thus, in areas of Sb mines, preferential accumulation has been observed in roots [41]. But with other species, and even sometimes in other work, greater accumulation has been obtained in the leaves [6,11,19], and the process depends on the presence of other elements that may interfere with the absorption and transport of Sb, as is the case with Sb-mining soils and *Dittrichia viscosa* [6,11]. The controversy around this point may be resolved by using hydroponic media to which different amounts of Sb have been added [16,42–44]. In this way, a fuller and more accurate study can be made of the process induced by Sb and its accumulation under totally controlled conditions.

The presence of Sb in soils implies its absorption by plants, with the consequent development of a heavy metal stress that alters the plants' physiological processes. In this work, we studied the effect of different levels of Sb on the growth, defence system redox reactions, degree of lipid peroxidation, non-enzymatic antioxidant content, photosynthetic pigments and photosynthetic efficiency, and on the mRNA expression of the CuZn SOD and GST in sunflower plants grown hydroponically with different inputs of Sb in the culture medium. We also studied the location of different ROS and RNS in intact roots of those plants using specific fluorescent probes.

## Material and methods

### Plant material

Seeds of sunflower (*Helianthus annuus*, L., cv. Safira) were surface sterilized for 15 min in 10% sodium hypochlorite solution (40 g L^-1^), rinsed several times with distilled water, and before their germination were imbibed in distilled water, aerated, and agitated for 2 h at room temperature. After imbibition the seeds were germinated in a plastic container (30 × 20 × 10 cm) filled with a sterilized perlite mixture substrate wetted with Hoagland solution, at 27°C, in the dark, for 48 h. After germination, the seedlings were cultivated for five days at 27°C with 85% relative humidity, and constant illumination under photosynthetic photon flux density (350 μmol m^-2^s^-1^).

After 7 days, the plants were grown in hydroponic culture in lightweight polypropylene trays (20 × 15 × 10 cm; 4 plants per container) and the same environmental conditions (except for relative humidity, 50%). The plants were treated with a basal nutrient solution composed of 4 mM KNO_3_, 3 mM Ca(NO_3_) _2_ 4H_2_O, 2 mM MgSO_4_ 7H_2_O, 6 mM KH_2_PO_4_, 1 mM NaH_2_PO_4_ 2H_2_O, 10 μM ZnSO_4_ 7H_2_O, 2 μM MnCl_2_ 4H_2_O, 0.25 μM CuSO_4_ 5H_2_O, 0.1 μM Na_2_MoO_4_ 2H_2_O, 10 μM H_3_BO_3_, and 20 μM NaFeIII-EDTA. For the Sb treatment, the basal solution was supplemented with KSb(OH)_6_ to final concentrations of 0.00 (control), 0.50, 0.75, and 1.00 mM Sb. Each cultivation solution was adjusted to pH 5.8, continuously aerated, and changed every 4 days. The plants were exposed to the Sb for 17 days.

Plants of each treatment were divided into roots and shoots which were washed with distilled water, dried on filter paper, and weighed to obtain the fresh weight (FW). Half of the roots and shoots from each Sb treatment were dried in a forced air oven at 70°C for 24 h to obtain the dry weight (DW) and the subsequent analysis of the concentration of Sb. The other half of the roots and leaves were used for the biochemical analyses.

### Elemental content analysis

The plant material, roots and leaves, of the control and Sb treatments was harvested and rinsed with distilled water. After 72 h of drying at 70°C, the root and leaf material was crushed in a marble ceramic mill. The elemental composition was measured by inductively coupled plasma mass spectrometry (ICP-MS, model) in accordance with Lehotai et al. [45].

### Chlorophyll and carotenoid contents and photochemical efficiency

The chlorophyll and carotenoid contents of the leaves were determined at the end of each trial. About 0.125 g of fresh leaves were incubated in 10 mL methanol for 24 h in the dark. The concentrations of chlorophylls and carotenoids were measured spectrophotometrically (Shimadzu UV1603) at A_666_ and A_653_. The total chlorophyll and carotenoid content was calculated as described by Wellburn [46].

For the determination of photosynthesis, leaves at the end of each Sb treatment were adapted in the dark for 10 min, and then the maximum photochemical efficiency (*F*_*V*_*/F*_*M*_) was recorded by a handheld fluorometer (Chlorophyll Fluorometer, OS-30p, Opti-Sciences).

### Determination of phenolic contents

Phenolics, flavonoids and phenylpropanoid glycosides were extracted from the plant material (roots or leaves) by homogenization with methanol, chloroform, and 1% NaCl (1:1:0.5). The homogenate was filtered and centrifuged at 3200 *g* for 10 min. Total phenolic content was assayed quantitatively by A_765_ with Folin-Ciocalteu reagent according to the method of Singleton et al. [47], and the result was expressed as μg of caffeic acid g^-1^ FW.

Phenylpropanoid glycosides (PPGs) were determined by a colorimetric method (A_525_) based on estimating an *o*-dihydroxycinnamic derivative using the Arnow reagent as described in Gálvez et al. [48]. The concentration was calculated on the basis of the standard curve of 3,4-dihydroxyphenylalanine, and expressed as μg verbascoside g^-1^ FW.

Total flavonoid content was measured colorimetrically following the method described by Kim et al. [49]. The total flavonoid content was calculated using the standard rutin curve and expressed as μg of rutin g^-1^ FW.

### Lipid peroxidation

Lipid peroxidation was determined by measuring malondialdehyde (MDA) formation using thiobarbituric acid (TBA). Briefly, 0.25 g plant material was homogenized with 2.5 mL of solution containing 0.25% TBA and 10% TCA. The mixture was incubated at 95°C for 30 min. The reaction was stopped by immersing the tubes in ice, filtering, and centrifuging at 8800 *g* for 10 min. The MDA was determined in the supernatant at A_532_—A_600_. The MDA concentration was calculated using an ε = 155 mM^-1^ cm^-1^, and expressed as μmol MDA g^-1^ FW [50].

### Oxidant and antioxidant enzyme activities

Plant material (0.5 g mL^-1^) was homogenized at 4°C in 50 mM phosphate buffer, pH 6.0. The homogenate was filtered and centrifuged at 39 000 *g* for 30 min at 4°C, the pellet was discarded, and the supernatant was filtered and collected as an enzyme extract. The protein content was determined by the method of Bradford [51].

The O_2_^.-^ generating activity was assayed spectrophotometrically by measuring the oxidation of epinephrine to adrenochrome at A_480_ (ε = 4.020 mM^-1^ cm^-1^) [52], [53]. The reaction mixture contained 1 mM epinephrine in acetate buffer 25 mM, pH 5.0.

Superoxide dismutase activity (SOD, EC 1.15.1.1) was determined as A_560_ in 50 mM phosphate buffer pH 7.8, 0.1 mM EDTA, 1.3 μM riboflavin, 13 mM methionine, and 63 μM NBT [54]. A unit of SOD is defined as the amount of enzyme required to cause 50% inhibition of NBT reduction.

Peroxidase activity (POX, EC 1.11.1.7), POX, was measured at A_590_ (ε = 47.6 mM^-1^ cm^-1^) [55], with the reaction mixture containing 3.3 mM DMAB and 66.6 μM MBTH in 50 mM phosphate buffer pH 6.0. A unit of POX is defined as the amount of enzyme required to cause the formation of 1 nmol DMAB-MBTH (indamine dye) per minute at 25°C, pH 6.0. The Ascorbate peroxidase activity (APX EC 1.11.1.11) was spectophotometrically determined by a decrease in A_290_ (ε = 2.8 mM^-1^ cm^-1^) for 3 min in 100 mM potassium phosphate buffer (pH 7.5), 0.5 mM ascorbate, and 0.2 mM H_2_O_2_ at 25°C [56].

Glutathione reductase activity (GR EC 1.6.4.2) was assayed by following the oxidation of NADPH at 340 nm (ε = 6.22 mM^-1^ cm^-1^) for 3 min [57]. The reaction medium contained 0.1 M potassium phosphate buffer (pH 7.5), 0.5 mM EDTA, 0.5 mM glutathione oxidized (GSSG), 0.2 mM NADPH, and 150 μL of protein extract in a volume of 1.5 mL.

Dehydroascorbate reductase activity (DHAR EC 1.6.4.2) was assayed by following the oxidation of NADPH at 340 nm (ε = 6.22 mM^-1^ cm^-1^) for 1 min [57]. The reaction medium contained 0.1 M potassium phosphate buffer (pH 7.5), 1 mM glutathione oxidized (GSSG), 0.1 mM NADPH, and 150 μL of protein extract in a volume of 1.5 mL.

GSNOR activity was assayed spectrophotometrically at 25°C by monitoring the oxidation of NADH at 340 nm for 3 min, as described by Sakamoto et al. [58]. The extracts were incubated in an assay mixture containing 20 mM Tris-HCl (pH 8.0), 0.5 mM EDTA, and 0.2 mM NADH, and the reaction was started by adding GSNO (Calbiochem) to the mixture at a final concentration of 400 μM. The activity was expressed as nmol NADH oxidized min^-1^ mg^-1^ protein (ε = 6.22 mM^-1^ cm^-1^).

Polyphenol oxidase activity (PPO, EC 1.14.18.1) was determined as described by Thipyapong et al. [59]. PPO activity was recorded by measuring the absorbance as A_390_ at 30°C, in a reaction medium composed of the enzyme extract, 100 mM phosphate buffer, Triton X-100, 30 μM caffeic acid. A unit of PPO is defined as the amount of enzyme required to cause a Δ A_390_ of 0.001 units min^-1^.

### Determination of total ascorbate and glutation

Plant material (1 g mL^-1^) were homogenized at 4°C in 5% metaphosphoric acid, in a porcelain mortar. The homogenate was centrifuged at 20000 g for 20 min at 4°C, and the supernatant was collected for determination of ascorbate and glutathione.

Total ascorbate and total glutathione were determined according De Pinto et al. [60]. Total ascorbate was determined by reduction of DHA to AsA and the concentration of DHA was estimated from the difference between total ascorbate pool (AsA + DHA) and AsA. The ascorbate pool is determined at 525 nm. The glutathione pool was assayed measurement the change in absorbance at 412 nm for 1 min. GSH was estimated as the difference between the amount of total glutathione (GSH + GSSG) and that of GSSG.

### Detection and visualization of O_2_^.-^, H_2_O_2_ and RSNOs

Samples of control and Sb-treated fresh roots were incubated for 30 min at 37°C in darkness, with 30 mM DCF-DA (for peroxide accumulation), and 15 mM DHE (for superoxide accumulation) in 10 mM Tris-HCl, pH 7.4, and washed twice for 10 min each in the same buffer [61]. After rinsed, whole roots (non fixed) slices of the different root zones were placed on a microscope slide and examined by fluorescence microscopy (Axioplan-Zeiss microscope). As negative controls, roots were pre-incubated before adding the probes in darkness for 60 min, at 25°C, with 1 mM ascorbate (peroxide scavenger) or 1 mM TMP (superoxide scavenger). For RSNOs detection, samples control and Sb-treated fresh roots were incubated for 60 min at 25°C in darkness, with 10 mM NEM prepared in ethanol, and then were washed three times (for 15 min each) in 10 mM Tris-HCl, pH 7.4. Then, the intact roots were incubated with 10 μM Alexa-Fluor 488 Hg-link phenylmercury for 60 min, at 25°C, in darkness [62]. Finally, the roots were washed three times in the same buffer (for 15 min each). After rinsed, whole roots (non fixed) slices of the different root zones were placed on a microscope slide and examined by fluorescence microscopy (Axioplan-Zeiss microscope). Parameters for fluorescence microscopy were identical for all experiments and control samples were always included.

For the root sections, samples of control and Sb-treated fresh roots (incubated in DCF-DA, or DHE, or Alexa-fluor 488) preserved in Tris-HCl (pH 7.4) were embedded in 2.5% agarose. The resulting agarose blocks were then glued to a metal block and submerged in a Tris-HCl bath. Transverse sections (150 μm thickness) of each sample were then performed with a Leica vibratome. Individual sections were collected with a brush, transferred to slides with a small amount of buffer and examined by fluorescence microscopy (Axioplan-Zeiss microscope).

Images were processed and analyzed using the ImageJ program and fluorescence intensity was expressed as arbitral units (AU). At least five roots were tested under each experimental condition and five independent repeats were analyzed.

### RNA isolation and semi-quantitative RT-PCR

Total RNA was isolated using the "Isolate II RNA Plant Kit" (Bioline) according to the manufacturer's instructions. Each extraction was replicated at least three times. After extraction, the concentration of the extract was determined by biophotometry (Eppendorf, Hauppauge, NY, USA), calculating the A_260_/A_280_ ratio. First-strand cDNA was synthesized using "High Capacity cDNA Reverse Transcription Kits" (Applied Biosystems): 2.0 μL of 10 x RT buffer, 0.8 μL of 25 x dNTP mix (100 mM), 2 μL 10 x RT random primers, 1.0 μL of MultiScribe™ reverse transcriptase, 1.0 μL RNase inhibitor, 3.2 μL nuclease-free H_2_O, and 10 μL of RNA. The reaction was done in a thermocycler (Eppendorf, Hauppauge, NY, USA) with a first stage at 25°C for 10 min, followed by a stage at 37°C for 120 min, and a final stage at 85°C for 5 min, obtaining single-stranded cDNA.

Semi-quantitative reverse-transcription PCR amplification of actin cDNA was chosen as control. In addition, PCR amplification was applied to identify CuZn-SOD I (chloroplastidial), CuZn-SOD II (cytosolic), and Glutathione-S-Transferase (GST). The PCR reaction was performed using the "BioTaq DNA Polymerase" kit (Bioline), with BIOTAQ polymerase, and using the selected pairs of oligonucleotides. The volume used for the PCR was 50 μL containing 5 μL of 10 x NH_4_ reaction buffer, 3 μL of 50 mM MgCl_2_, 0.5 μL 100 mM dNTP mix, 1.5 μL of each of the different pairs of primers (5 μM), 1 μL of BIOTAQ polymerase, and RNase- and DNAse-free water. The PCR conditions were: 95°C for 3 min 30 s, 55°C for 30 s, and a final extension at 72°C for 30 s for each kb, for 35 cycles in each PCR reaction. The PCR products were developed in 1% (w/v) agarose gel with ethidium bromide. The bands were quantified using a gel doc system together with a high sensitivity charge-coupled device (CCD) camera. The gene-specific primers used were (5´-GCTCCTAAGCCGCTTACGGTTGTCG-3´) and (5´-CACGCCATCGGCATTGGCAATTATG-3´) for CuZn-SOD I (accession AJ786257), and (5´-TGCAGTACCATCTTCGCCTACTGTGACA-3´) and (5´-TGCAGTACCATCTTCGCCTACTGTGACA-3´) for CuZn-SOD II (accession AJ786258), and (5´-GTCGGTTCCAACCTTCGT-3´) and (5´-GGGCAACAAACATTACACACTCC-3´) for GST (accession EC:2-5-1-18). As controls, we used (5´-TTCTTCTCTCCCCCAATTTCAGCCA-3´) and (5´-AAACTCGGGGCACCTGACCGCT-3´) for actin (accession 10355).

### Statistical analysis

The data presented are the means ± SD of at least 10 replicates obtained from three independent experiments. The data were analysed statistically by the Mann-Whitney U-test.

## Results

### Growth and biomass production

Cultivating the plants with Sb reduced both root and stem growth, as well as the production of biomass (Table 1). There was also development of foliar chlorosis. The length of the primary root decreased in all treatments with Sb in the nutrient solution. The greatest reduction (27%) was with the 1 mM Sb concentration. Stem length growth, however, was not significantly affected by any of the Sb concentrations used. There was a strong decrease in fresh weight and dry weight of both root and stem with all Sb concentrations in the nutrient medium. The effect was stronger with increasing Sb. Thus, total biomass production was reduced by approximately 63% relative to the controls. The decrease in biomass production became more pronounced with 1 mM, with the total dry weight and fresh weight of the plant decreasing by factors of 2.6 and 2.4 compared to the control values, respectively.

**Table 1:** Effect of Sb treatments on Sb accumulation in plant tissues (μg g^-1^ DW), transfer factor (TF), fresh weight (g), dry weight (g) and total longitude (cm). Data from 5 independent experiments, each one carried out in triplicate (different letters indicate significant differences at p<0.05, Mann-Whitney U-test).

Sb treatment	Net Sb in the plant tissue		TF*	Fresh Weight (g)		Dry Weight (g)		Longitude (cm)	
(mM)	(μg g^-1^ DW)								
	Roots	Leaves		Roots	Shoots	Roots	Shoots	Roots	Shoots
0.00	0.00a	0.00a	0.00a	3.90±0.78a	9.73±2.03a	0.168±0.03a	0.664±0.012a	33.18±5.77a	17.45±1.39a
0.50	3263.9±86.1b	491.2±22.0b	0.15b	2.58±0.93b	8.82±1.78a	0.117±0.02b	0.648±0.010a	28.64±1.79a	16.60±1.40a
1.00	9780.9±112.4c	1216.7±101.9c	0.12b	1.57±0.43c	3.53±0.78b	0.079±0.04c	0.267±0.009b	24.30±2.68b	17.20±1.11a

* TF: * TF: the ratio between the concentration of Sb in the leaves and in the roots.

The increase in Sb concentration in the nutrient solution induced a significant increase in the Sb content of the tissues of both roots and leaves (Table 2). This increased accumulation occurred preferentially in the roots, where the values of this element were several times greater than that obtained in the leaves. In both cases, the accumulation was dependent on the dose of Sb supplied. The translocation factor was very low, clearly indicative of how this compound accumulates preferentially in the roots. In addition, the absorption and accumulation of Sb altered the content of other mineral elements such as Mg, Fe, Cu, and Zn. Thus, we observed decreases in the roots content of Fe and Zn. The foliar content decreases in the content of Mg, Fe and Zn, but the Cu content increases. The rest of the mineral elements underwent no significant changes in their content.

**Table 2:** Effect of Sb treatments on Magnesium (Mg), Iron (Fe), Copper (Cu) and Zinc (Zn) concentrations in roots and leaves. Data from 5 independent experiments, each one carried out in triplicate (different letters indicate significant differences at p<0.05, Mann-Whitney U-test).

Sb treatment	Mg (mg g^-1^ DW)		Fe (μg g^-1^ DW)		Cu (μg g^-1^ DW)		Zn (μg g^-1^ DW)	
(mM)	Roots	Leaves	Roots	Leaves	Roots	Leaves	Roots	Leaves
0.00	10.7±0.05a	6.50±0.10a	929.75±8.50a	194.76±3.66a	24.82±0.24	16.98±0.03a	65.14±1.20a	68.62±2.80a
0.50	21.2±0.06b	5.00±0.08b	775.34±6.79b	136.15±1.90b	23.80±0.17	21.91±0.04b	45.15±1.11b	51.11±1.56b
1.00	12.9±0.04a	4.00±0.09c	203.35±3.50c	107.34±1.60c	25.00±0.21	24.55±0.02c	33.78±2.36c	39.90±2.10c

### Photosynthetic pigment content and maximum photosynthetic efficiency

The concentrations of Sb used significantly reduced the amounts of photosynthetic pigments (Table 3). Total chlorophylls, including chlorophyll a and, above all, chlorophyll b, were reduced in concentration in the leaves. The reduction was by up to 43% for chlorophyll a and 51% for chlorophyll b relative to the controls. The 0.50 mM Sb treatment presented chlorophyll levels similar to those of the controls. In contrast, the carotenoid content presented no significant alterations. Only with the highest concentration was there a decrease of approximately 10%, although this was not significant. With regards to the maximum photosynthetic efficiency (Fv/Fm) (Table 3), there was a clear response to increases in Sb, similar to what was observed in the chlorophyll content. The values of Fv/Fm fell from 0.785 in the controls and similar for the low concentration of Sb to 0.606 in 1.00 mM Sb.

**Table 3:** Effect of Sb treatments on total chorophyll a and b, carotenoids, and *F*_*V*_*/F*_*M*_ (Photosyntetic efficiency). Data from 5 independent experiments, each one carried out in triplicate (different letters indicate significant differences at p<0.05, Mann-Whitney U-test).

Sb treatment (mM)	Pigments content (μg g^-1^ FW)			*F*_*V*_*/F*_*M*_
	Chla	Chlb	Car	
0.00	2011.73±117.93a	1111.68±126.79a	204.64±21.47a	0.785±0.008a
0.50	1919.84±90.15a	927.04±96.52a	211.24±29.51a	0.781±0.026a
1.00	1161.27±117.21b	511.00±78.79b	182.13±31.47a	0.606±0.018b

### Total phenols, flavonoids, and phenylpropanoid glycosides

**Fig 1:**
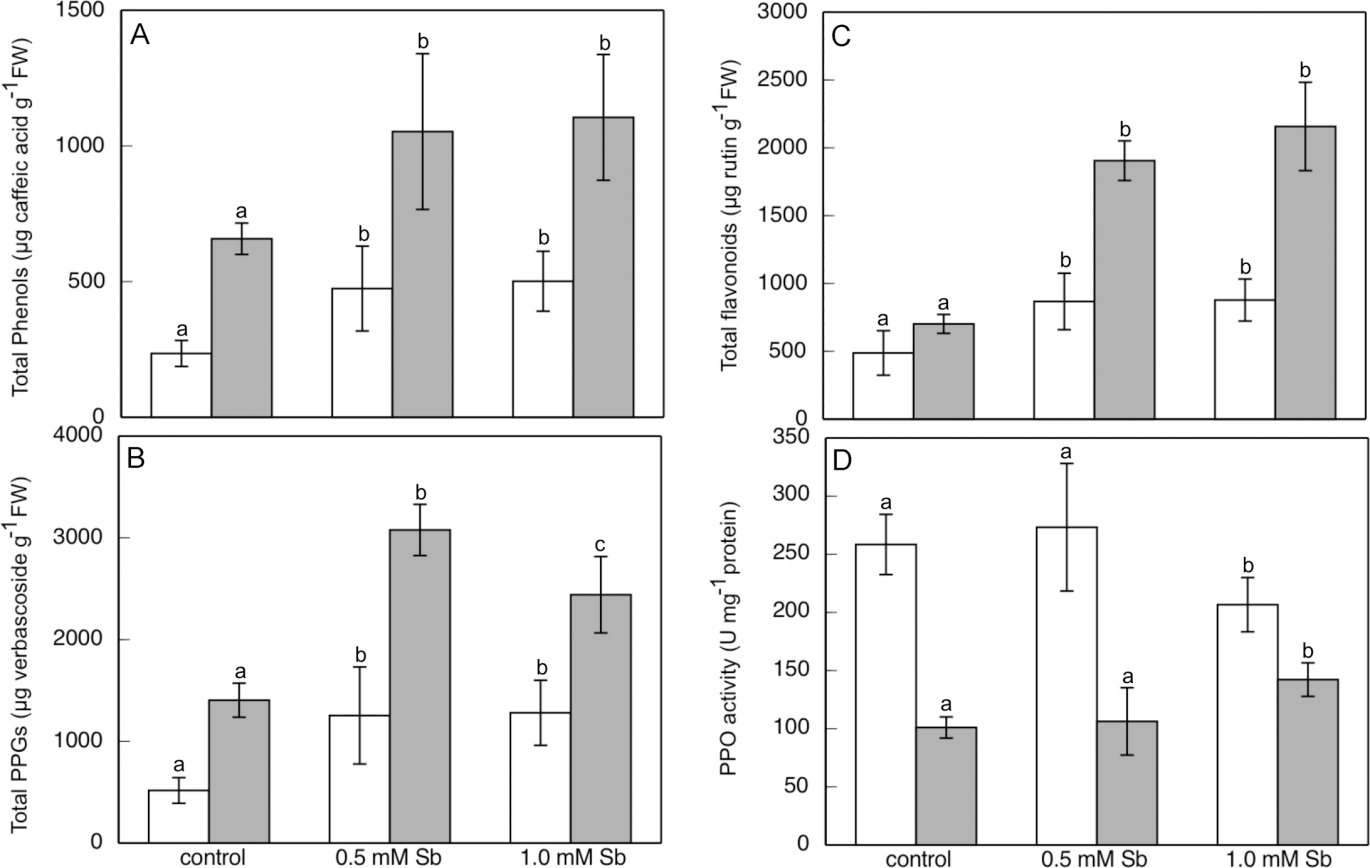
Effect of Sb on the total phenols content (A), phenilpropanoid glycosides (B), flavonoids (C), and PPO activity (D) in roots (□) and leaves (■) of *H*. *annuus* plants. Data from 5 independent experiments, each one carried out in triplicate (different letters indicate significant differences at p<0.05, Mann-Whitney U-test).

The effect of Sb on total phenols (Fig 1A) was to increase their production and accumulation in both roots and leaves. The increase was similar for all concentrations, and there were no significant differences between them in either of the organs studied. Thus, relative to the controls, there were increases of approximately 100% in roots and 50% in leaves in response to the treatments with Sb. Regarding the total phenylpropanoid glycosides content (Fig 1B), the production and accumulation of these compounds increased in response to the Sb toxicity. In roots, the effect was much like that observed for total phenols, and the increases were similar for all concentrations of Sb. In leaves, however, the greatest increase in these compounds was in response to the lowest concentration (0.50 mM Sb), being slightly less at the highest concentration. Finally, the flavonoids (Fig 1C) presented a fluctuating behaviour. Their content was greater in both organs in response to treatment with Sb, with the increase being by a factor of 3 in leaves with the 1 mM Sb concentration. The PPO activity (Fig 1D) in the roots was not significantly affected by Sb. There were even slight decreases in this activity with 1.0 mM Sb. The same was the case with the leaves, although in this case they presented a greater PPO activity.

### Peroxidation of lipids and production of superoxide anion

**Fig 2:**
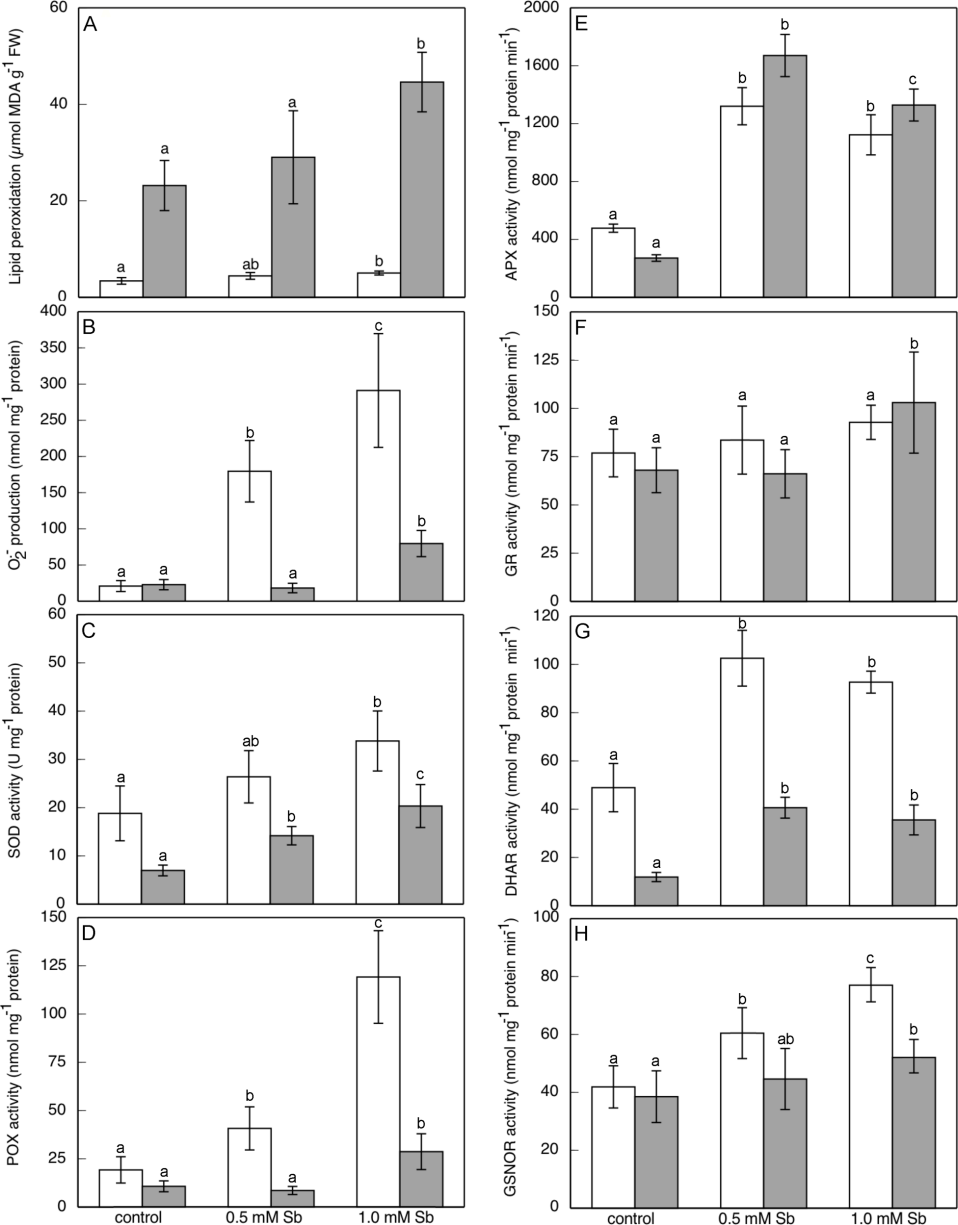
Effect of Sb on lipid peroxidation (A) and, on the superoxide production (B), SOD (C), POX (D), APX (E), GR (F), DHAR (G), and GSNOR (H) activities in roots (□) and leaves (■) of *H*. *annuus* plants. Data from 5 independent experiments, each one carried out in triplicate (different letters indicate significant differences at p<0.05, Mann-Whitney U-test).

The plants treated with 1.0 mM of Sb showed significant increases in lipid peroxidation (MDA) in both roots and leaves (Fig 2A). In leaves, the values duplicated those of the controls, but in roots, the increase was smaller. With the 0.5 mM Sb concentration, the levels of lipid peroxidation measured were lower than those with 1.0 mM Sb.

The treatments with Sb induced a strong and significant increase in the production of O_2_^.-^ (Fig 2B), which initiates the oxidative shock. In roots, the increases were dose dependent, being by factors of 8 and 14 for 0.5 mM and 1.0 mM Sb, respectively. In leaves, the effect was similar but more moderate for the highest concentration, but the 0.5 mM Sb concentration did not alter the O_2_^.-^ levels.

### Antioxidant activities and components of the ascorbate/glutathione cycle

There were strong increases in SOD activity (Fig 2C) in both root and leaf in response to exposure to Sb. For 1.0 mM Sb, the increases were by factors of 2 in roots and 3 in leaves. The effect was dose dependent, with intermediate values for the 0.5 mM Sb treatment.

There were large increases in POX activity in the roots in response to both Sb treatments (Fig 2D). The behaviour was different in the leaves, however. There was no change in POX activity for the 0.5 mM Sb treatment, and the increase in the case of 1 mM Sb was comparatively smaller than that in the roots. The APX activity increase with both Sb treatments (Fig 2E), specially with 0.5 mM Sb. The increase in this activity is greather in leaves than in roots. These results show opposite behavior between the two peroxidase determined activities. The POX activity is the most important in roots, while on the contrary in leaves the APX activity could be responsible of removing the excess H_2_O_2_ and the lipid peroxides.

With regard to the activities of the ascorbate/glutathione cycle, GR (Fig 2F) only presented increased activity in leaves for the 1.0 mM Sb treatment. In roots, there were no significant alterations of this activity.

The DHAR activity (Fig 2G) in roots increased with the 1.0 mM Sb treatment, but not with the lower concentration. On the contrary, in leaves with both Sb concentrations, the values were very similar to or lower than the controls, evidence for a decline in this activity.

Finally, for the GSNOR activity (Fig 2H), Sb induced a stronger response in the roots, with an increase of more than 80%, and somewhat less at the lower concentration (48%). In leaves, the increases were smaller, approximately 18% and 35%, respectively.

The AsA/DHA and GSH/GSSG contents are important parameters in the cellular redox status (Table 4). In the roots, a slight increase was observed in AsA and DHA content, but it was not significant. However, in the leaves with 1.0 mM Sb, the AsA content reached values that were much higher than the controls and DHA decreased significantly. The total content of AsA+DHA in roots increased with the Sb treatments, reaching very similar values in all of them. In leaves on the contrary, no alterations were observed with respect to the control.

**Table 4:** Effect of Sb treatments on total ascorbate (AsA+DHA), reduced ascorbate (AsA), dehydroascorbate (DHA), and the ratio ascorbate/dehidroascorbate (AsA/DHA); and total glutathione (GSH+GSSG), reduced glutathione (GSH), oxidized glutathione (GSSG), and the ratio reduced glutathione/oxidized glutathione (GSH/GSSG) in roots and leaves of *H*. *annuus*. Data from 5 independent experiments, each one carried out in triplicate (different letters indicate significant differences at p<0.05, Mann-Whitney U-test).

**Sb treatment (mM)**	**AsA + DHA (nmol g**^**-1**^ **FW)**		**AsA (nmol g**^**-1**^ **FW)**		**DHA (nmol g**^**-1**^ **FW)**		**AsA / DHA (nmol g**^**-1**^ **FW)**	
	**Roots**	**Leaves**	**Roots**	**Leaves**	**Roots**	**Leaves**	**Roots**	**Leaves**
0.00	285.90±70.38a	1724.28±147.89a	118.22±48.30a	158.73±15.83a	167.68±51.04a	1565.55±132.74a	0.614±0.154a	0.102±0.008a
0.50	343.53±76.48a	1764.17±153.50a	138.34±34.78a	261.32±82.47b	204.89±50.54a	1502.85±235.25a	0.757±0.201a	0.174±0.051a
1.00	350.12±68.71b	1667.98±149.90a	153.68±27.82b	374.33±74.68b	196.44±36.96a	1293.65±187.10b	0.757±0.217a	0.301±0.110b
**Sb** **treatment(mM)**	**GSH + GSSG (nmol g**^**-1**^ **FW)**		**GSH (nmol g**^**-1**^ **FW)**		**GSSG (nmol g**^**-1**^ **FW)**		**GSH / GSSG (nmol g**^**-1**^ **FW)**	
	**Roots**	**Leaves**	**Roots**	**Leaves**	**Roots**	**Leaves**	**Roots**	**Leaves**
0.00	8.32±1.98a	19.16±2.11a	5.84±1.74a	8.86±0.22a	2.48±0.42a	10.3±1.92a	2.390±0.690a	0.882±0.163a
0.50	6.48±2.40ab	15.91±8.76ab	5.08±1.76a	10.17±4.99a	1.40±0.73b	5.74±4.31b	4.682±1.060b	3.090±1.570b
1.00	5.05±1.80b	12.23±1.40b	3.70±1.82b	6.38±1.34b	1.35±0.23b	5.85±1.45b	2.677±1.150a	1.170±0.470a

With respect to GSH content, in both roots and leaves, reductions were observed in the treatment with 1.0 mM Sb but not with 0.5 mM Sb. The amount of GSSG decreased in both roots and leaves with the Sb treatments, the values being similar for the two concentrations. The total GSH+GSSG content in both organs decreased in response to Sb, the more so the greater the Sb concentration.

The cellular redox status of AsA/DHA and GSH/GSSG (Table 4) responds to the Sb-induced phytotoxicity. The AsA/DHA ratio in roots increased in response to the Sb treatments. There was no dependence on the concentration, with similar values of this ratio being obtained for all the concentrations of Sb used. In leaves on the contrary, the AsA/DHA ratio remained unchanged at the lowest concentrations but increased at the highest. With respect to the GSH/GSSG ratio, this presented a more fluctuating behaviour. In both roots and leaves, this ratio's highest values were obtained with 0.5 mM Sb, while the rest of the treatments gave intermediate values, in some cases similar to the controls.

### Visualization of the accumulation of ROS and RSNOs

**Fig 3:**
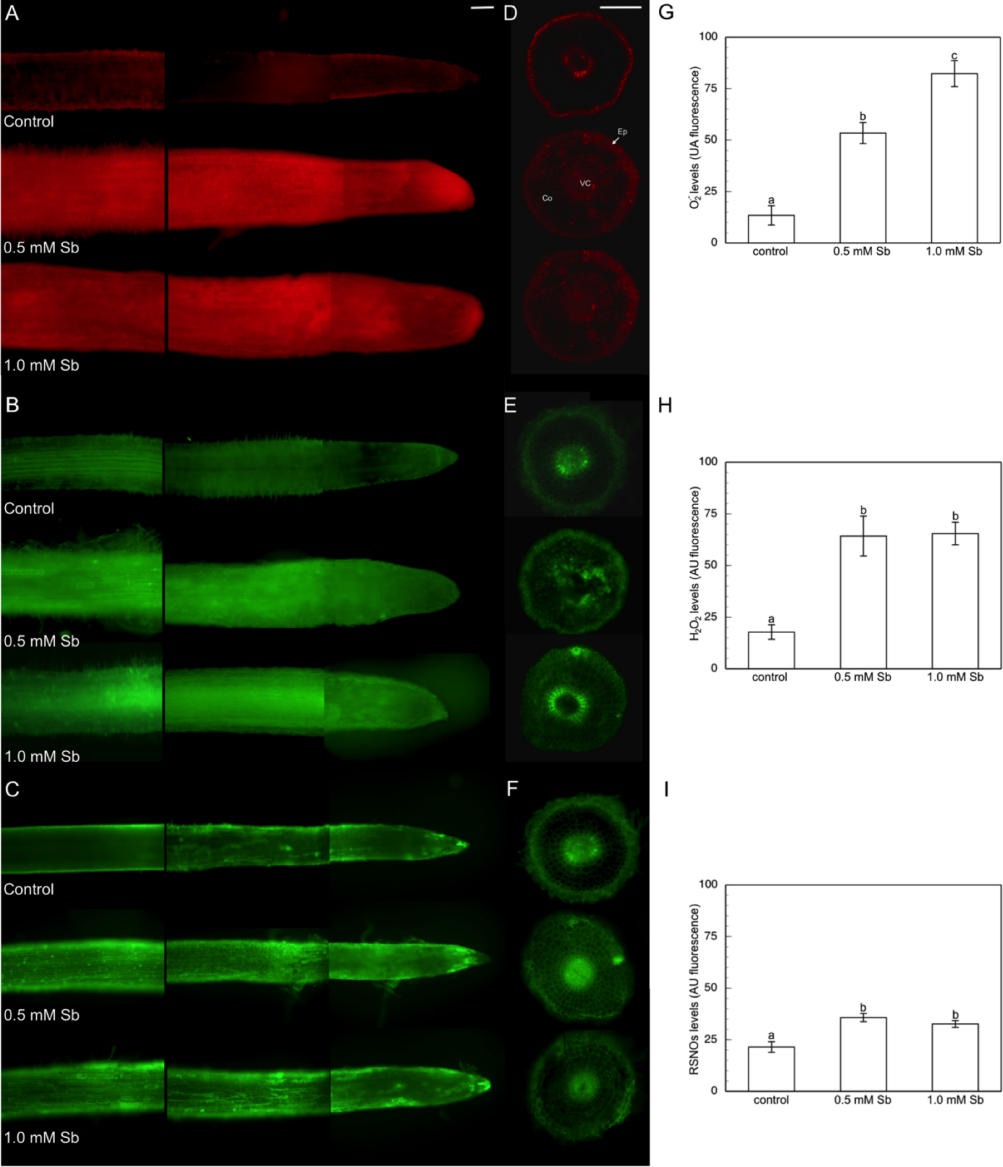
Detection of superoxide anion, hydrogen peroxide and RSNOs production in longitudinal section (A, B, C), in cross-section at level of elongation zone (D, E, F), and the average fluorescence intensity levels quantified in arbitrary units (G, H, I), respectively. At least 5 roots were tested for each experimental condition and 5 independent repeats were analyzed. Ep (epidermis), Co (cortex), VC (vascular cylinder). Bar: 200 μm.

There was a clear increase in the accumulation of ROS and RSNOs in roots in response to Sb (Fig 3). The accumulation of O_2_^.-^ depended on the Sb concentration, with the greatest accumulation in roots treated with 1.0 mM Sb, although the accumulation was much greater than the control value even with 0.5 mM Sb (Fig 3A, 3D and 3G). One observes in the cross-sectional images made at the level of the zone of root elongation that this increase in O_2_^.-^ affected both the vascular cylinder (VC) and the epidermal cells, where there was already some accumulation in the control roots, and especially the cells of the cortex at 1 mM Sb. With the lowest Sb concentration, the increase was not generalized to all cells, but restricted to the VC and epidermis. The accumulation of H_2_O_2_ and RSNOs presented a different behaviour to the foregoing. The accumulation observed was very similar for both Sb concentrations (Fig 3B, 3E, 3H, 3C, 3F and 3I, for H_2_O_2_ and RSNOs, respectively), in some cases being higher with 0.5 mM Sb than with 1 mM Sb, although not significantly. As with the accumulation of O_2_^.-^, under control conditions, these reactive species accumulated preferentially in the VC and epidermis, and with less intensity in some cells of the cortex. In contrast, in response to Sb toxicity, the accumulation was more generalized, extending to all cell types of roots.

### Analysis of the mRNA expression of the CuZnSOD and GST

**Fig 4:**
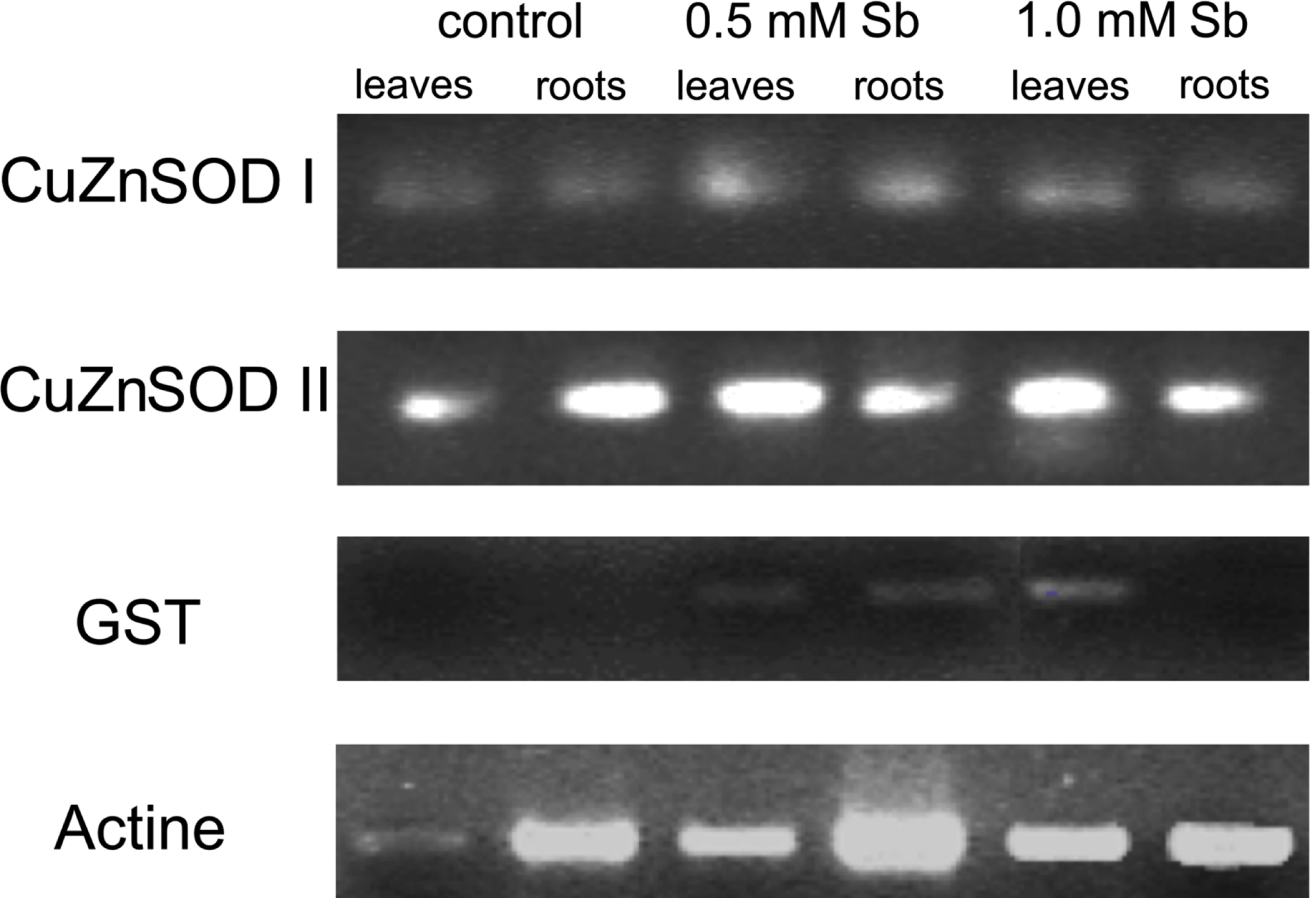
Effect of Sb on the mRNA expression of the isoenzymes CuZn SOD I, CuZn SOD II, and GST in sunflower leaves and roots using semiquantitative reverse transcription-PCR on total RNA isolated.

Semi-quantitative reverse-transcription PCR amplification of mRNA corresponding to the CuZn-SOD and GST enzymes was performed for sunflower plants exposed to Sb (Fig 4). As can be observed, the expression pattern of the CuZn-SOD I and CuZn-SOD II isozymes is similar in roots and leaves of both control plants and those exposed to Sb, although the expression bands are stronger and clearer in the Sb-exposed plant material, except for the CuZnSOD II in roots. The CuZnSOD II expression was higher in control roots than in the ones exposed to Sb. This is not the case, however, for GST expression. While in roots this expression is observed in both control and Sb plants (although the expression bands are clearer in the latter), in leaves there is no band observed in the controls, a clear band is present in the Sb plants.

## Discussion

The decrease in biomass production due to Sb toxicity has been described in various plants [44,63–65] as also for other heavy metals such as Cd [66]. In all the cases, fresh weight, dry weight, and total biomass production were reduced, as also was the length of the root system. These findings are similar to the results described by Pan et al. [19] for maize, and are characteristic symptoms of heavy metal toxicity [67,68]. Also observed was preferential accumulation of the absorbed Sb in the roots, consistent with very low TF values in non-Sb-accumulating plants [42,44,65,69]. This reduced root development may be due to an alteration in the elongation of the cells located in the root elongation zone, as can be seen in the longitudinal fluorescence microscopy images (data no show).

The effect of high doses of Sb on the concentration of other elements has been studied in several plants. Thus, in wheat, Shtangeeva et al. [70] report reduction of the Ca, K, Na, and Cu contents, and Feng et al. [20] report decreases in Ca, Mg, Fe, Mn, Cu, and Zn. Our results coincide in part with those, except for Cu which we found to increase. The increase in the amount of Cu is consistent with the results reported by Feigl et al. [39], although they were in response to Zn toxicity.

With respect to the effect of Sb on the total content of chlorophylls and carotenoids, our results are similar to those obtained by Pan et al. [19] and Xue et al. [71] who describe a clear decrease in the biosynthesis of chlorophylls in response to Sb, with a smaller alteration of the carotenoid content. This decrease in the content of photosynthetic pigments could also be related to the lower leaves concentration of Fe and Mg in the Sb-treatments. Studying Cd toxicity, Zayneb et al. [72] obtain a similar response in the content of compounds of this type, with the reduction in the case of carotenoids only being significant for the highest Cd concentration. The lower photosynthetic efficiency observed in sunflower plants treated with Sb may be due to an alteration in the electron transport in PSII which acts to dissipate energy [73]. The reduction in the content of photosynthetic pigments also affects the antioxidant capacity in these plants, which would cause greater oxidative damage. Despite the low transport and accumulation of Sb to leaves, it has a very strong effect on the photosynthetic pigment content and photosynthetic efficiency.

The increase observed in the phenolic compounds, especially the flavonoids, in response to Sb toxicity is evidence of their protective effect against oxidative damage. This effect could be due to changes these compounds produce in the membranes, altering their permeability and thereby reducing the diffusion of ROS. Their capacity to interact with membrane phospholipids may act to maintain membrane integrity [35,36]. In addition, they could be involved in O_2_^.-^ and H_2_O_2_ elimination processes [74,75]. Apoplastic peroxidases act together with phenols and Asc to eliminate H_2_O_2_ [37]. The activity of flavonoids and peroxidases constitutes an effective system for the elimination of H_2_O_2_. Apoplastic and vacuolar peroxidases could form oxidized phenolic radicals, involved in the direct elimination of ROS and participating in the control of the redox status through reactions that form DHA, which, through the action of DHAR, is reduced to Asc. Also the PPGs increase in response to the stress induced by Sb. These phenolic compounds could be modulate the antioxidant enzyme activities, as well as the direct scavenging of ROS and RNS [76–78].

The increase observed in O_2_^.-^ production in both roots and leaves is a clear response to the oxidative stress induced by Sb. This strong increase occurred even despite the high level of SOD activity that both organs presented as a consequence of the Sb treatment. This increased activity was comparatively greater in leaves than in roots, which could explain the smaller increase in the amount of O_2_^.-^ detected in the leaves. Nonetheless, in neither organ was this increase in SOD activity sufficient to avoid overaccumulation of O_2_^.-^. These results are similar to those described by other workers [41,72] who have observed a strong increase in SOD activity in response to Sb toxicity, both in leaves and in roots. They are also consistent with those described by Feigl et al. [39] in *B*. *napus* roots treated with Zn toxicity resulting in increases in the amount of superoxide anion and in SOD activity. However, those same authors describe a different response in *B*. *juncea*, with a decrease in O_2_^.-^ and increases in SOD activity, showing that the same stress can result in different responses in different species. Results that are contrary to ours are described by Feng et al. [63] and Pan et al. [19], with a progressive decrease in SOD activity in response to increasing Sb concentrations. A consequence of the increase in O_2_^.-^ production and SOD activity is the increased accumulation in the roots of O_2_^.-^ and H_2_O_2_ observed in the Sb treatments as against the controls. This is seen in the fluorescence images in which the accumulation of ROS extends to all the cells of the root, without being restricted to zones of more active growth and in processes of maturation. On the contrary, in the control roots, the amount of ROS is much smaller, being also restricted to the epidermis and VC. The accumulation of O_2_^.-^ is dependent on the concentration of Sb, but not that of the H_2_O_2_ which is similar in both concentrations. The O_2_^.-^ level showed a significant increment, which is associated with a strong increase in SOD activity. However, the elevated SOD activity was not sufficient to remove the excess of O_2_^.-^. These results are very similar to those described in response to stresses induced both by pathogens and elicitors [79] and [80] and by heavy metals [39]. These accumulations are consistent with the data for SOD and O_2_^.-^ production activity described above.

The peroxidase activities (POX and APX) were also considerably greater in both organs. The POX activity is greater with the 1 mM Sb treatment, while for the APX activity it is with 0.5 mM Sb. This is a consequence of the increased SOD activity resulting in greater amounts of H_2_O_2_. Also, they were consistent with the higher levels of lipid peroxidation in a response similar to that described by Benhamdi et al. [41]. This result coincides with that described in response to Sb in different species for peroxidase activities in response to Sb [41,43,64,71]. Also, Feng et al. [81] and Zayneb et al. [72] obtained higher lipid peroxidation levels despite of the increased antioxidant activity in response to other heavy metals. On the contrary, Chai et al. [65] obtained results that show sharp declines in SOD, peroxidase and catalase activities.

The POX activity was also considerably greater in both organs, especially with the 1 mM Sb treatment. This is a consequence of the increased SOD activity resulting in greater amounts of H_2_O_2_, in a response similar to that described by Benhamdi et al. [41]. Despite this increase in POX activity however, there were higher levels of lipid peroxidation in response to the stress induced by Sb, especially in the leaves. It is possible that the great activation of POX that was observed in roots was able to partially prevent this peroxidative damage, although it was not enough to avoid it altogether. This result coincides with that described in response to Sb in different species [41,43,64,71]. Also, Feng et al. [78] and Zayneb et al. [72] obtained higher lipid peroxidation levels despite increased antioxidant activity in response to other heavy metals. On the contrary, Chai et al. [65] obtained results that show sharp declines in SOD, peroxidase and catalase activities.

With respect to the behaviour of enzymatic activities related to the ascorbate/glutathione cycle, this differed between roots and leaves. In the roots, GR activity did not change, but DHAR activity increased significantly. In the leaves, on the contrary, both GR and DHAR activity increase in response to the stress induced by Sb. These activities are key to the regeneration of reduced glutathione and ascorbate, and the data we obtained in leaves are similar to those described by Singh et al. [81] in response to treatment with As. Feng et al. [16] describe a decrease in GR activity in response to Sb, although their work considered the whole plant.

The total AsA content increased in both organs. The DHA content was not significantly affected by the treatment with 0.5 mM Sb, but with 1 mM Sb there was a decrease in leaves. This led to the total AsA+DHA content increasing only in roots, with no change in leaves, indicative of a greater capacity for antioxidant response in the former. This increase was not correlated with an increase in the amount of GSH and GSSG. Instead the results showed a decrease in the total contents of both GSH and GSSG in both organs. These declines led to the GSH+GSSG content decreasing significantly. Singh et al. [81] observed an increase in the amount of DHA and a decrease in that of AsA, although the total AsA+DHA content increased. While this result is similar in part to ours in terms of total content, but it differs in the separate behaviour of AsA and DHA which was contrary to our findings. Those workers also describe an increase in the amount of GSH and GSSG, whereas in our case there was a strong decrease in both in response to Sb, especially in the case of GSSG whose content fell by approximately 40% in both organs in response to both concentrations of Sb. Srivastava et al. [66] report that exposure of rice plants to Cd toxicity induces a decrease in the amount of GSH in both roots and leaves, as in our case with 1 mM Sb. However, while in their work the amount of GSSG increased, and the GSH/GSSG ratio fell considerably, our data are in the opposite sense, with decreases in the GSSG content and increases in the GSH/GSSG ratio. Other workers [16] describe alterations in the content of GSH and GSSG together with increases in GR activity which coincide with our observations despite being in response to a different toxicity. This decrease in GSH and GSSG content, especially for 1 mM Sb, could be related to greater formation of RSNOs. The increase in these compounds in response to Sb is evidenced by their greater accumulation as observed in the fluorescence images, and by the increase in GSNOR activity. The accumulation of RSNOs is produced by all root cell types, and similar for both Sb treatments. This increase in RSNOs has been described in responses to pathogen attack [80], with their accumulation being principally in the cells of the infected root.

In the plants subjected to Sb stress, we observed increased GST expression, possibly indicating the participation of this group of enzymes in Sb detoxification. Together with the greater amount of GSH observed in leaves, this enhanced GST expression may be involved in that detoxification via the formation of GSH-metal complexes and by catalysing the binding of GSH to toxic compounds [82–85]. The amount of GSSG, however, decreases. This is reflected in a raised GSH/GSSG ratio, while the AsA/DHA ratio does not change. The result is an imbalance between the two cycles. The observed increase in both GST expression and the amount of GSH in response to Sb toxicity are indicative of their participation in mechanisms of Sb tolerance. These results would seem to be coherent with those reported for *Arabidopsis* in which the overexpression of GST increases tolerance to Al and Cu with low levels of peroxidation ([86] and to As, Cd, and Cr [87], and for poplar with respect to Hg [88].

Moreover, Sb stress increases SOD activity, but lipid peroxidation is only increased significantly at 1 mM Sb. This may be due to the observed increases in expression of the GSTs and CuZn-SOD which may act to catalyse the reduction of hydroperoxides. This would help to avoid Sb-induced oxidative damage, maintaining protein functionality and redox homeostasis [89]. Indeed, we observed that Sb treatment significantly increased the activity of DHAR (belonging to the group of GSTs), especially in roots, which would contribute to increased antioxidant activity through the formation of AsA. Sb induces increases in enzymatic (SOD, POX, APX, DHAR and GSNOR) and non-enzymatic (phenols, flavonoids, PPGs, AsA and only in leaves for the GSH) systems in sunflower. A similar increase is observed in the expression of CuZnSOD I and II, and GST.

In conclusion, our results indicate participation of phenolic compounds, the antioxidant systems, and the AsA/GSH cycle in the processes of defence against the oxidative stress induced by Sb. The phenolic compounds and the antioxidant systems both show increased activity. In addition to the effects of flavonoids on membranes, this participation may involve their action together with the enzymatic antioxidant systems and the ascorbate/glutathione redox cycle in the processes of ROS detoxification and redox homeostasis. The observed imbalance between the redox pool of AsA+DHA and GSH+GSSG might indicate direct action of Sb on the -SH groups, especially dithiols [90], for which it has a great affinity. This union would provoke disequilibrium of this system, reducing the intracellular pool of GSH+GSSG, and thus altering the plant's antioxidant capacity. Despite the increase in the antioxidant response, Sb induces major oxidative damage which has a direct influence on the plant's growth, affecting the accumulation of certain mineral elements and the photosynthetic capacity, besides altering the cellular redox balance.
